# The relationship between neuromyelitis optica spectrum disorder and autoimmune diseases

**DOI:** 10.3389/fimmu.2024.1406409

**Published:** 2024-06-27

**Authors:** Jie Lin, Binbin Xue, Jia Li, Dewei Xie, Yiyun Weng, Xu Zhang, Xiang Li, Junhui Xia

**Affiliations:** ^1^ Department of Neurology, First Affiliated Hospital of Wenzhou Medical University, Wenzhou, China; ^2^ Department of Anesthesiology, First Affiliated Hospital of Wenzhou Medical University, Wenzhou, China

**Keywords:** Neuromyelitis optica sectrum disorders, autoimmune disease, annualized relapse rate (ARR), autoimmune antibodies, prognosis

## Abstract

**Objective:**

There have been reports of neuromyelitis optica spectrum disorder (NMOSD) coexisting with connective tissue disorders. The objective of this study was to describe the characteristics of NMOSD coexisting with autoimmune diseases (AID).

**Methods:**

This retrospective study evaluated NMOSD patients with and without AID. The enrolled patients had at least one attack, with duration of more than 1 year. Data on the demographics, clinical features, and laboratory findings were assessed. The Poisson model was used to investigate the risk factors associated with the annualized relapse rate (ARR), whereas the Cox model was used to evaluate the risk factors for the first relapse.

**Results:**

A total of 180 patients (154 women and 26 men) with NMOSD were identified: 45 had AID and 135 did not. Female patients had a higher prevalence of concomitant AID (*p* = 0.006) and a greater relapse rate within the first year. There were no statistically significant differences in the characteristics of patients. Kaplan–Meier analysis revealed that NMOSD patients with seropositive aquaporin 4 antibodies (AQP4-Ab; log-rank: *p* = 0.044), had a shorter time to relapse. Patients seropositive for AQP4-Ab (HR = 2.402, 95%CI = 1.092–5.283, *p* = 0.029) had a higher risk of suffering a first relapse, according to the Cox model. Patients with and without AID showed a similar declining tendency in terms of change in ARR throughout the first 5 years of the disease. The ARR was greater in the first year [incidence rate ratio (IRR) = 1.534, 95%CI = 1.111–2.118] and the first 2 years (IRR = 1.474, 95%CI = 1.056–2.058) in patients with coexisting AID diagnosis prior to the NMOSD onset.

**Conclusions:**

Patients with NMOSD with coexisting AID had similar characteristics when compared with those without AID. NMOSD patients with AID diagnosed before onset had a higher risk of relapse in the early stage of the disease.

## Introduction

Neuromyelitis optica spectrum disorder (NMOSD) is a central nervous system (CNS) inflammatory disorder characterized by recurrent optic neuritis (ON), transverse myelitis (TM), brain syndrome, and brainstem syndrome (1). Pathogenic antibodies, such as anti-aquaporin 4 antibodies (AQP4-Ab), target the endfeet of astrocytes, resulting in a series of clinical syndromes (2). As is known, B-lymphocyte-mediated immunity plays a vital role in the pathogenesis of autoimmune disorders. Patients with NMOSD have coexisting connective tissue disorders (CTDs), such as systemic lupus erythematosus (SLE) and Sjögren’s syndrome (SS), as reported in several studies (3–9). The annualized relapse rate (ARR), the number of attacks, and the Expanded Disability Status Scale (EDSS) at the last follow-up were not statistically significant between NMOSD patients with or without CTD (6). In another study, the first attack NMOSD patients with coexisting CTDs had higher recurrence rates, more recurrences, and short remission (9). In addition, NMOSD patients with coexisting myasthenia gravis (MG) and autoimmune thyroid disease (AITD) are not uncommon.

However, it is still unknown whether the coexistence of AID affects the progression of patients with NMOSD. Immunosuppressive treatment strategies for these patients have still not been specified. The objective of this study was to investigate the demographic characteristics of these patients, the clinical processes, and the potential role of AID in NMOSD.

## Methods

### Patients

The medical records of patients with NMOSD were reviewed in our hospital from 2012 to March 2022. All patients had at least one attack, with a duration of more than 1 year. NMOSD was diagnosed according to the 2015 International Consensus Diagnostic (IPND) criteria for NMOSD (10), while CTD was diagnosed by rheumatologists based on CTD-related criteria [i.e., SLE, SS, and reactive arthritis (RA)] (11–13). Neurologists diagnosed MG according to the consensus on MG (14). AITD was diagnosed by endocrinologists. Medical records including the presence of AQP4-Ab, autoreactive antibodies (antinuclear antibodies, ANA), anti-Sjögren’s syndrome-related antigen A (SSA) antibodies (SSA), anti-SSB antibodies (SSB), anti-dsDNA antibodies (ds-DNA), anti-neutrophil cytoplasmic antibodies (ANCA), anti-mitochondrial antibodies (AMA), anti-histone antibodies (AHA), anti-cardiolipin antibodies (ACA), anti-Scl7 antibodies (Scl7), anti-Smith antibodies (SM), anti-PM/Scl antibodies (Pmscl), anti-Jo-1 antibodies (Jo-1), anti-nucleosome antibodies (ANuA), and anti-thyroid-associated antibodies (ATA). Patients without complete medical records and those with a disease duration of less than 1 year were excluded from the study.

The Ethics Committee of the First Affiliated Hospital of Wenzhou Medical University approved this study.

### Data collection

The clinical data of patients with NMOSD were collected, including selected demographic characteristics (age of onset, gender, and disease duration), clinical manifestations (optic neuritis, myelitis, and other lesions), number of attacks, the serostatus of AQP4-Ab and of the autoimmune antibodies, maintenance treatment, and previous medical history such as the condition of autoimmune diseases. AQP4-Ab was examined using fixed cell-based indirect immunofluorescence tests. HEK293 cells transfected with either the M1 isoform of aquaporin 4 (AQP4) were used. The abovementioned autoantibodies were assessed in the clinical and immunology laboratory of our hospital.

To evaluate the different clinical symptoms at onset and relapse, the following clinical attacks were classified: isolated ON, isolated myelitis, other lesions, and multiple lesions.

The ARR was calculated as the number of relapses per year. A relapse was defined as follows: 1) new neurological symptoms lasting more than 24 h and 2) worsening neurological symptoms lasting longer than 24 h without other etiology.

Immunosuppressive treatments (ISTs) were retrospectively reviewed. There were 186 patients with NMOSD who were prescribed glucocorticoids (GCs), azathioprine (AZA), mycophenolate mofetil (MMF), rituximab (RTX), and inerizumab. The remaining 11 patients did not receive prophylaxis.

### Statistical analysis

Statistical analysis was performed using the Statistical Package for the Social Sciences (version 23.0; IBM, Armonk, NY, USA). Categorical data were presented as percentages. Continuous data were presented as the mean and standard deviation (SD) and ranked data as the median and interquartile range (IQR). The Mann–Whitney *U* test or Student’s *t*-test was applied for quantitative data, while the chi-squared test or Fisher’s exact test was utilized for qualitative data. Poisson regression was used to analyze the possible factors related to the attacks in the first year, the first 2 years, and the first 5 years during the disease course. The time to the first relapse after onset was analyzed with Kaplan–Meier analysis. The Cox hazards model was used to evaluate the risk factors related to the first attack. *P*-values of <0.05 were considered statistically significant. *P*-values that did not show statistical significance were not presented.

## Results

### Characteristics

A total of 230 NMOSD inpatients from our hospital were reviewed. Of these, 50 patients were excluded due to: 1) incomplete clinical data or loss to follow-up (*n* = 32) and 2) the disease duration being less than 1 year (*n* = 18). A total of 180 patients (154 women and 26 men) who met the inclusion criteria were enrolled in this cohort. There were 157 and 12 patients with NMOSD who were seropositive and seronegative for AQP4-Ab, respectively. The serostatus of AQP4-Ab in the remaining 11 patients was unknown. The enrolled patients suffered attacks ranging from 1 to 22. A total of 45 patients with NMOSD had coexisting autoimmune diseases (AID): SS (*n* = 23), SLE (*n* = 8), SS+SLE (*n* = 3), ACA syndrome (*n* = 1), undifferentiated arthritis (*n* = 2), RA (*n* = 1), AITD (*n* = 3), MG+APS (autoimmune polyglandular syndrome) (*n* = 1), APS+AITD (*n* = 1), SS+APS (*n* = 1), and autoimmune hepatitis+SLE+SS (*n* = 1). There were 15 patients with AID diagnosed before the onset of NMOSD.

The demographics and clinical characteristics of NMOSD patients with and without AID are summarized in **Table 1**. Female patients with NMOSD had a higher occurrence of AID (*p* = 0.006). A high frequency of seropositive autoimmune antibodies (AIAs) (*p* < 0.001), including ANA (*p* < 0.001), Ro52 (*p* < 0.001), SSA (*p* < 0.001), SSB (*p* < 0.001), ds-DNA (*p* < 0.001), AMA (*p* = 0.006), anti-AHA (*p* < 0.001), and ACA (*p* = 0.024), was observed in NMOSD patients with AID. However, no statistical significance was detected in the seropositivity to AQP4-ab, age of onset age, duration of disease, ARR, the first year of ARR (ARR_1_), the number of total attacks, and the attacks of ON, myelitis, and other lesions, as well as the type of first attack (**Table 1**).

**Table 1:** Characteristics of neuromyelitis optica spectrum disorder (NMOSD) patients with and without autoimmune disease (AID).

	AID (*n* = 45)	Non-AID (*n* = 135)	*p*-value
Gender			0.006
Women, *n*	44 (97.8%)	110 (81.6%)	
Men, *n*	1 (2.3%)	25 (18.4%)	
Age of onset (years)	41.44 ± 14.06	42.60 ± 15.28	
Seropositive for AQP4-Ab	41 (97.6%)	116 (91.3%)	
IST	43 (95.6%)	107 (79.3%)	0.010
Disease duration (years)	6.50 ± 4.79	7.69 ± 5.43	
Annual relapse rate	0.59 ± 0.29	0.55 ± 0.37	
Onset type			
ON	18 (40.0%)	46 (34.1%)	
Myelitis	12 (26.7%)	47 (34.8%)	
Other lesions	8 (17.8%)	27 (20.0%)	
Multiple lesions	7 (15.6%)	15 (11.1%)	
Attacks, mean±SD	3.40 ± 2.54	3.52 ± 3.00	
ON, mean±SD	1.31 ± 1.52	1.28 ± 1.85	
Myelitis, mean±SD	1.96 ± 2.09	1.89 ± 2.12	
Other lesions, mean±SD	0.38 ± 0.65	0.53 ± 1.06	
Seropositive for autoimmune antibodies, *n*	45 (100%)	91 (67.4%)	<0.001
ANA	45 (100%)	68 (50.4%)	<0.001
Ro52	34 (75.6%)	21 (15.6%)	<0.001
SSA	30 (66.7%)	16 (11.9%)	<0.001
SSB	14 (31.1%)	6 (4.4%)	lt;0.001
ds-DNA	9 (20.0%)	1 (0.7%)	<0.001
AMA	7 (15.6%)	5 (3.7%)	0.006
Jo-1	1 (2.2%)	2 (1.5%)	
ANCA	3 (6.7%)	5 (3.7%)	
AHA	12 (26.7%)	10 (7.4%)	<0.001
ACA	7 (15.6%)	7 (5.2%)	0.024
Scl70	2 (4.4%)	0	
SM	3 (6.8%)	1 (0.7%)	
Pmscl	0	3 (2.2%)	
ANuA	3 (6.7%)	2 (1.5%)	
ATA	9 (20%)	21 (15.6%)	

AQP4-Ab, anti-aquaporin 4 antibodies; IST, immunosuppressive treatments; ON, optic neuritis; ANA, antinuclear antibodies; SSA, anti-Sjögren’s syndrome-related antigen A antibody; SSB, anti-Sjögren’s syndrome-related antigen B antibody; AMA, anti-mitochondrial antibodies; ANCA, anti-neutrophil cytoplasmic antibodies; AHA, anti-histone antibodies; ACA, anti-cardiolipin antibodies; SM, anti-Smith antibodies; Pmscl, anti-PM/Scl antibodies; ATA, anti-thyroid-associated antibodies; ANuA, anti-nucleosome antibodies; Jo-1, anti-Jo-1 antibodies; ds-DNA, anti-double-stranded DNA antibodies.

According to previous studies, patients with NMOSD who are seropositive for AIAs but cannot be diagnosed as having AID were more common than those with other CNS inflammatory disorders. To understand the characteristics of these patients, we divided them into three groups (as described in **Table 2**): patients with AID (*n* = 45), patients who are seropositive for AIAs but could not be diagnosed with AID (*n* = 101), and patients who are seronegative for AIAs (*n* = 48). No statistical differences in the clinical parameters, including onset age, disease duration, ARR, onset lesion, and accumulated attacks, were observed (**Table 2**).

**Table 2:** Characteristics of neuromyelitis optica spectrum disorder (NMOSD) patients with autoimmune disease (AID) who are seropositive and seronegative for autoimmune antibodies (AIA).

	AIA seropositive	AIA seronegative	AID	P1	P2
Gender					
Women, *n*	74 (81.3%)	36 (81.8%)	44 (97.8%)	0.027	
Men, *n*	17 (18.7%)	8 (18.2%)	1 (2.2%)		
Age of onset (years)	44.14 ± 14.08	39.41 ± 17.24	41.44 ± 14.06		
Seropositive for AQP4-Ab (*n* = 128)	80 (94.1%)	36 (85.7%)	41 (97.6%)		
IS	74 (81.3%)	33 (75.0%)	43 (95.6%)	0.026	<0.05
Disease duration	7.65 ± 5.77	7.77 ± 4.71	6.50 ± 4.79		
Annual relapse rate	0.55 ± 0.35	0.56 ± 0.42	0.59 ± 0.29		
Onset type					
ON	29 (31.9%)	17 (38.6%)	18 (40.0%)		
Myelitis	31 (34.1%)	16 (36.4%)	12 (26.7%)		
Other lesions	19 (20.9%)	8 (18.2%)	8 (17.8%)		
Multiple lesions	12 (13.2%)	3 (6.8%)	7 (15.6%)		
Attacks, mean±SD	3.54 ± 3.20	3.48 ± 2.56	3.40 ± 2.54		
ON, mean±SD	1.27 ± 1.72	1.30 ± 2.11	1.31 ± 1.52		
Myelitis, mean±SD	1.97 ± 2.28	1.77 ± 1.75	1.96 ± 2.09		
Other lesions, mean±SD	0.54 ± 1.13	0.52 ± 0.93	0.38 ± 0.65		
Seropositive for AIA, *n*	91 (100%)	0	45 (100%)	<0.001	
ANA	68 (74.7%)	0	45 (100%)	<0.001	<0.05
Ro52	21 (23.1%)	0	34 (75.6%)	<0.001	<0.05
SSA	16 (17.6%)	0	30 (66.7%)	<0.001	<0.05
SSB	6 (6.6%)	0	14 (31.1%)	<0.001	<0.05
ds-DNA	1 (1.1%)	0	9 (20.0%)	<0.001	<0.05
AMA	5 (5.5%)	0	7 (15.6%)	0.011	
Jo-1	2 (2.2%)	0	1 (2.2%)		
ANCA	5 (5.5%)	0	3 (6.7%)		
AHA	10 (11.0%)	0	12 (26.7%)	<0.001	<0.05
ACA	7 (7.7%)	0	7 (15.6%)		
Scl70	0	0	2 (4.4%)		
SM	1 (1.1%)	0	3 (6.7%)		
Pmscl	3 (3.3%)	0	0		
ANuA	2 (2.2%)	0	3 (6.7%)		
ATA	21 (23.1%)	0	9 (20.0%)	0.003	

P1 denotes inter-group values. P2 are values for seropositive antibodies and the coexistence of AID.

AQP4-Ab, anti-aquaporin 4 antibodies; IST, immunosuppressive treatments; ON, optic neuritis; ANA, antinuclear antibodies; SSA, anti-Sjögren’s syndrome-related antigen A antibody; SSB, anti-Sjögren’s syndrome-related antigen B antibody; AMA, anti-mitochondrial antibodies; ANCA, anti-neutrophil cytoplasmic antibodies; AHA, anti-histone antibodies; ACA, anti-cardiolipin antibodies; SM, anti-Smith antibodies; Pmscl, anti-PM/Scl antibodies; ATA, anti-thyroid-associated antibodies; ANuA, anti-nucleosome antibodies.

The mean age of onset in women was earlier than that in men (*p* < 0.001), and women had a higher frequency of having coexisting AID (*p* = 0.002). Male patients with AID had a lower ARR_1_. No statistical significance was found in ARR; duration of disease; total number of attacks; number of episodes of ON, myelitis, and other lesions; treatment with immunosuppressants (IS); serostatus of AIA; and age of onset.

### Age of onset in NMOSD patients with AID

Patients with disease onset earlier than age 50 were classified as early-onset NMOSD (EO-NMOSD), while those with onset age 50 years or older were classified as late-onset NMOSD (LO-NMOSD) (15). Previous studies have indicated that the age of onset affects the clinical characteristics and prognostic outcomes of patients with NMOSD. In this study, 61 patients were identified as having EO-NMOSD, while the remaining 119 were identified as having LO-NMOSD. Patients with EO-NMOSD suffered more clinical attacks (3.91 ± 3.24 vs.. 2.67 ± 1.77, *p* = 0.001), particularly attacks of ON (*p* < 0.001). There was no significant difference in the number of myelitis and other lesions between the two groups. In addition, there were more female patients with EO-NMOSD than male patients (*p* = 0.001).

There were no statistically significant differences between the EO-NMOSD and LO-NMOSD groups in terms of seropositivity to AQP4-Ab, ISTs, concurrent AID (*p* = 0.210), AID diagnosis before NMOSD onset, and the serostatus of AIAs, ANA, Ro52, SSA, SSB, ds-DNA AMA, Jo-1, ANCA, AHA, ACA, Scl70, SM, Pmscl, ANuA, and ATA.

### Role of AID in NMOSD

**Figure 1:**
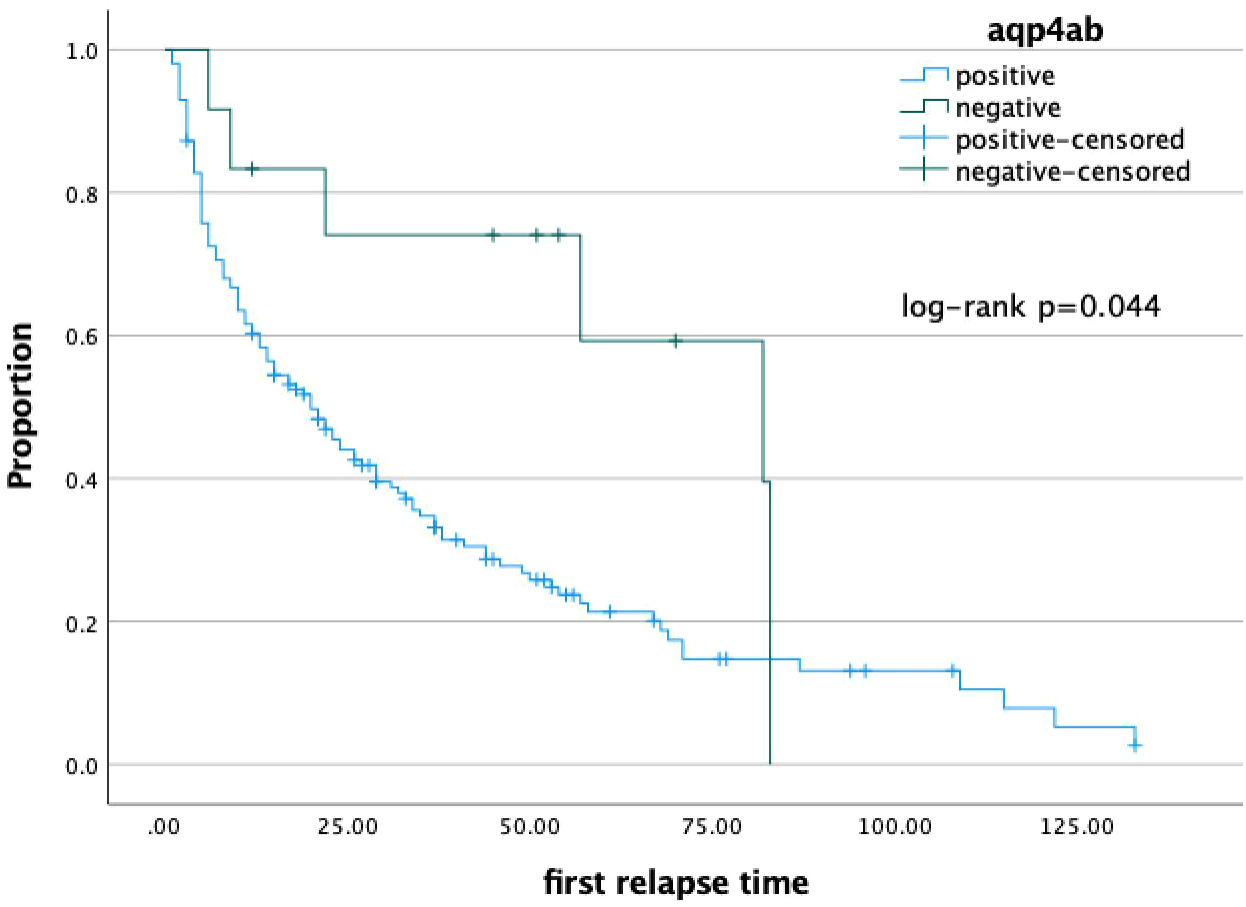
Kaplan-Meier analysis indicated NMOSD patients with seropositive AQP4-ab(A) (36.33±3.54 vs. 60.72±9.80 months, log-rank: p=0.044) had a shorter duration tp next relapse than seronegative patients, respectively.

The Kaplan–Meier survival analysis revealed that the time to first relapse in NMOSD patients seropositive for AQP4-Ab (36.33 ± 3.54 vs. 60.72 ± 9.80 months; log-rank: *p* = 0.044) (**Figure 1**) was earlier than that in seronegative patients. NMOSD patients with coexisting AID, as well as those with AID diagnosed before the onset of NMOSD, did not show statistically significant differences. These factors were then analyzed using Cox proportional hazards regression. It was found that patients with NMOSD seropositive for AQP4-Ab (HR = 2.402, 95%CI = 1.092–5.283, *p* = 0.029) had a higher risk of suffering the first relapse after onset.

**Figure 2:**
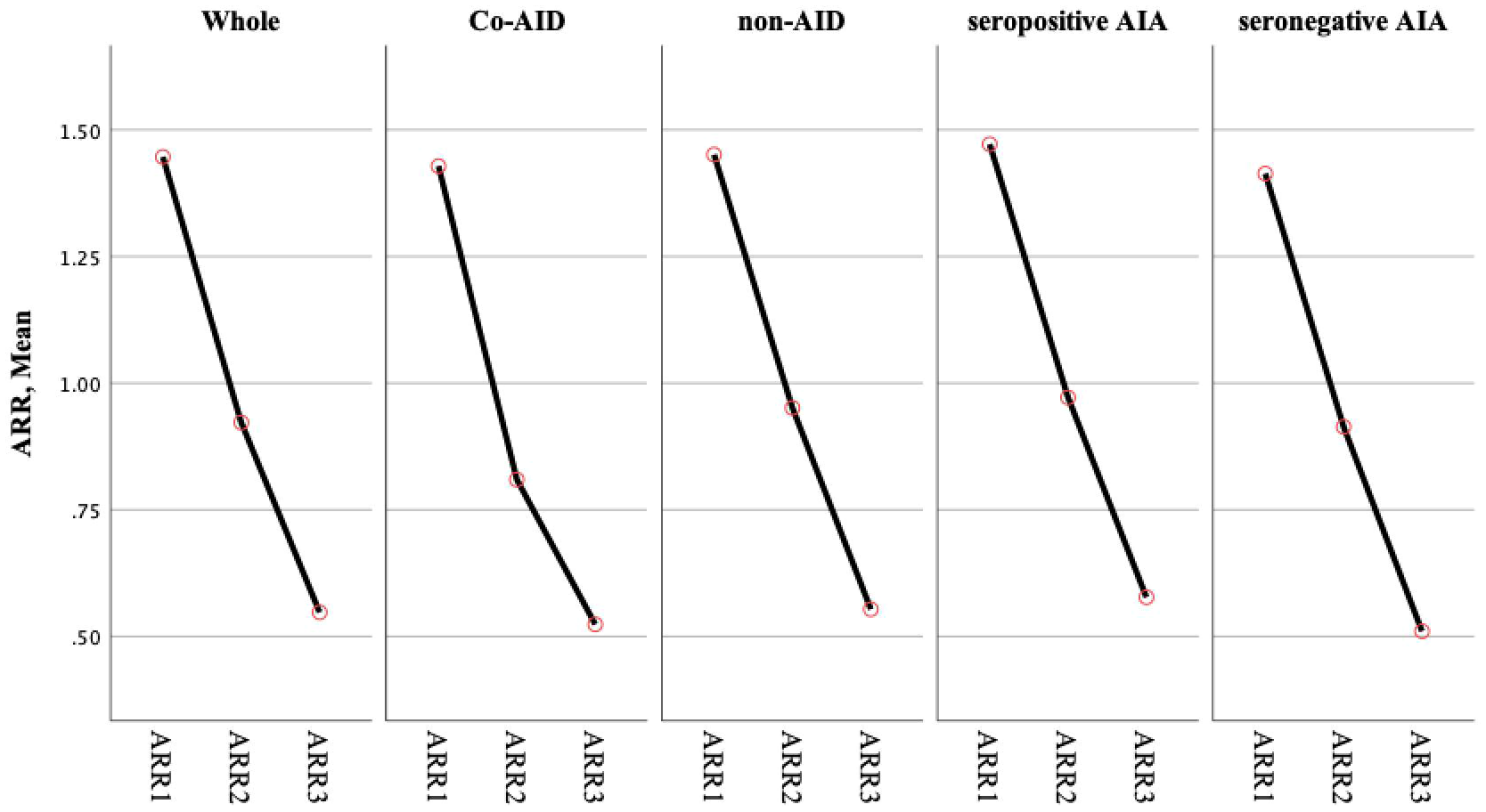
ARR showed the declined tendency in the whole cohort and subgroups. No statistically significant difference was observed in groups. ARR1: ARR of the first 1 year; ARR2: ARR of the first 2 years; ARR3: ARR of the first 5 years ARR.

To better understand the role of AID during NMOSD, data from the cohort with a disease duration of more than 5 years (*n* = 103) were further analyzed. The demographics and the characteristics of the AID (*n* = 21) and non-AID (*n* = 82) patients from this cohort did not show statistically significant differences. The ARRs of the first year (ARR_1_), the first 2 years (ARR_2_), and the first 5 years (ARR_5_) after onset in patients with AID were significantly reduced, which is consistent with the those of the whole cohort (*p* > 0.05) and in non-AID patients (*p* > 0.05) (**Table 3**, **Figure 2**). The Poisson regression model indicated that diagnosis of AID before the onset of NMOSD was associated with a higher risk of suffering more attacks in the first year of disease (IRR = 1.534, 95%CI = 1.111–2.118) and the first 2 years (IRR = 1.474, 95%CI = 1.056–2.058), while this effect was not sustained over 5 years (*p* > 0.05). Seropositivity to AQP4-Ab showed an increased IRR in the first 5 years (IRR = 1.864, 95%CI = 1.196–2.905, *p* = 0.006).

**Table 3:** Annualized relapse rates (ARRs) in patients with and without coexisting autoimmune disease (AID).

	AID			Non-AID			*p*
	First year	First 2 years	First 5 years	First year	First 2 years	First 5 years	
Cohort 1	*N* = 45			*N* = 135			
Attack, no	1.42 ± 0.58	–	–	1.44 ± 0.59	–	–	NS
ARR	1.42 ± 0.58	–	–	1.44 ± 0.59	–	–	NS
Relapse rate	37.8%	–	–	39.3%	–	–	NS
Cohort 2	*N* = 40			*N* = 118			
Attack, no	1.45 ± 0.60	1.65 ± 0.80	–	1.41 ± 0.57	1.78 ± 0.81	–	NS
ARR	1.45 ± 0.60	0.83 ± 0.40	–	1.41 ± 0.57	0.89 ± 0.40	–	NS
Relapse rate	41.0%	50.0%	–	37.3%	57.6%	–	NS
Cohort 3	*N* = 21			*N* = 82			
Attack, no	1.43 ± 0.60	1.62 ± 0.80	2.62 ± 1.24	1.45 ± 0.61	1.90 ± 0.87	2.77 ± 1.66	NS
ARR	1.43 ± 0.60	0.81 ± 0.40	0.52 ± 0.25	1.45 ± 0.61	0.95 ± 0.43	0.55 ± 0.33	NS
Relapse rate	38.1%	47.6%	81.0%	40.2%	62.2%	79.3%	NS

Cohort 1: disease duration of more than 1 year, but less than 2 years; cohort 2: disease duration more than 2 years, but less than 5 years; cohort 3: disease duration of more than 5 years. P-values are for inter-group.

NS, not significant.

## Discussion

The natural history of NMOSD patients with coexisting AID is not fully recognized. This study revealed that patients with coexisting AID have similar clinical characteristics to those without AID, regardless of whether the AID was diagnosed before or after the onset of NMOSD. Female patients more frequently had coexisting AID and had a high frequency of seropositive AIAs (4–9). SS was the most frequent comorbidity in NMOSD, consistent with previous epidemiological studies. Yang et al. reported that characteristics such as ARR and the number of attacks did not differ between NMOSD patients with and without CTDs (6). However, other studies have indicated that patients with NMOSD who are seropositive to AQP4-Ab and with coexisting CTDs have more relapses and higher relapse rates (9). It is known that the number of attacks taper off after a cluster of attacks following disease onset (1, 3, 16). We observed that patients with and without AID had significantly reduced ARR over time (*p* < 0.05). In addition, a stepwise declining trend in ARR was shown in the whole cohort and in subgroups, without statistically significant differences.

Six core clinical syndromes have been identified in NMOSD involving the optic nerve, spinal cord, area postrema, brainstem, diencephalon, and the cerebrum (1, 10). ON and TM are the most common manifestations in patients with NMOSD. Sentinel attacks involve ON and TM in more than 85% of affected adult NMOSD patients (1). The NMOSD patients with AID in our cohort had similar prevalence rates of ON and TM. As illustrated in **Table 1**, there were no statistical differences in the number of total attacks, ON, TM, and other lesions between the two groups. Patients with NMOSD had a higher relapse rate (76% vs. 48.86%), had more relapses, and had a short duration to the first relapse when the CTD was diagnosed before the onset of NMOSD (9). In our cohort, the patients with AID diagnosed before NMOSD had a higher risk of relapse in the first year and the first 2 years compared with non-AID patients, although the effect diminished over time. The natural history of NMOSD and the prescription of ISTs, including MMF and RTX, might account for this phenomenon.

AQP4-Ab are highly sensitive and specific for the diagnosis of NMOSD (2). The frequency of seropositivity to AQP4-ab did not show statistical differences between NMOSD patients with and without CTDs (4, 6, 9). The results from our study are consistent with those of previous studies. Seropositivity to AQP4-Ab is recognized as the risk factor related to disease relapse, even in cohorts of pregnant women and children (16–18). Unsurprisingly, seropositivity to AQP4-ab was the common risk factor for predicting relapse during the disease. Interestingly, it was found that patients seropositive for SM and Pmscl had a shorter duration to the first relapse after the disease onset.

B-lymphocyte-mediated immune response plays a vital role in the pathogenesis of SS, SLE, MG, APS, and undifferentiated connective tissue disease (UCTD) (14, 19–22). ISTs, including AZA, MMF, and RTX, have been proven effective in CTDs (19, 20, 22) and NMOSD (9, 17, 23, 24). Therapeutic challenges arise in NMOSD patients with coexisting AID; however, RTX, an anti-CD20 antibody, has shown efficacy in real-world practice (25–27). Recommendations for patients with NMOSD might be effective for those with coexisting AID. Five years after the onset of NMOSD, nearly 25% of untreated AQP4-Ab seropositive patients required wheelchair assistance due to accumulated disability (16). In a Chinese cohort, the EDSS did not show a marked difference between NMOSD patients with and without CTDs after a long-term follow-up (6, 9). Data from our cohort indicated that patients with AID diagnosed before the onset of NMOSD had a relapse tendency in the early stages of the disease, suggesting the need for more efficient ISTs in these patients during the early stage of NMOSD. B-cell deletion therapy, such as RTX, has been recognized as a more efficient IST compared with GCs, AZA, and MMF (9, 23, 28). With a deeper understanding of the pathogenesis of NMOSD, therapy with anti-IL6, anti-C5, and anti-CD19 have proven efficacy in patients with NMOSD (29–31). Patients with AID might benefit from the early use of highly effective drugs.

There are limitations in this study. A major limitation is that this study is retrospective, which could lead to potential biases; however, due to the rarity of NMOSD coexisting with AID, the data from our cohort are acceptable. Further data from studies with larger sample sizes across multiple centers and ethnic groups are needed.

## Conclusion

Patients with NMOSD with coexisting AID had similar characteristics to NMOSD patients without AID. NMOSD patients with AID diagnosed before disease onset had a higher risk of relapse in the early stage of the disease.

## Data availability statement

The original contributions presented in the study are included in the article/supplementary material. Further inquiries can be directed to the corresponding authors.

## Ethics statement

The studies involving humans were approved by The Ethics Committee of the First Affiliated Hospital of Wenzhou Medical University. The studies were conducted in accordance with the local legislation and institutional requirements. Written informed consent for participation was not required from the participants or the participants’ legal guardians/next of kin in accordance with the national legislation and institutional requirements.

## Author contributions

JLin: Conceptualization, Data curation, Formal analysis, Methodology, Project administration, Software, Writing – original draft, Writing – review & editing. BX: Data curation, Formal analysis, Methodology, Writing – review & editing. JLi: Data curation, Investigation, Resources, Writing – review & editing. DX: Data curation, Formal analysis, Methodology, Writing – original draft. YW: Data curation, Formal analysis, Investigation, Methodology, Writing – original draft. XZ: Conceptualization, Project administration, Supervision, Writing – review & editing. XL: Conceptualization, Formal analysis, Funding acquisition, Supervision, Writing – original draft, Writing – review & editing. JX: Conceptualization, Methodology, Project administration, Supervision, Writing – original draft, Writing – review & editing.
